# Pharmacist-Led Deprescribing Interventions in Older Adults: A Systematic Review of Medication Appropriateness, Patient Safety, and Clinical Outcomes

**DOI:** 10.7759/cureus.106525

**Published:** 2026-04-06

**Authors:** Esteban Zavaleta-Monestel, Jeaustin Mora-Jiménez, Luis Herrera-Jiménez, Andrea Chaves-Arroyo, Kevin Cruz Mora, Sebastián Arguedas-Chacón

**Affiliations:** 1 Department of Research, Hospital Clinica Biblica, San José, CRI; 2 Department of Research, Hospital Clínica Bíblica, San José, CRI; 3 Pharmacy, Hospital Clinica Biblica, San Jose, CRI; 4 Pharmacy, Hospital Clinica Biblica, San José, CRI

**Keywords:** aged adult, deprescribing, inappropriate prescribing, polypharmacy management, prescribing safety

## Abstract

Polypharmacy in older adults is associated with medication-related harm, including adverse drug events, drug-drug interactions, inappropriate prescribing, and hospitalization-related outcomes. This systematic review evaluated the effectiveness and safety of pharmacist-led deprescribing interventions in adults aged ≥65 years. The review was reported according to the Preferred Reporting Items for Systematic Reviews and Meta-Analyses (PRISMA) 2020 statement, and searches were conducted in PubMed, Embase, and Scopus for studies published from January 2020 to December 2024. Five studies met the inclusion criteria, including two randomized controlled trials and three quasi-experimental studies across inpatient, emergency department, and ambulatory settings. Pharmacist-led interventions were generally associated with reductions in potentially inappropriate medication use and improvements in medication appropriateness. Evidence regarding adverse drug events, hospital admissions/readmissions, mortality, and other major clinical outcomes remained limited and heterogeneous. Overall, pharmacist-led deprescribing appears feasible and may improve prescribing quality, but larger randomized studies with longer follow-up are needed to determine its effect on major clinical outcomes.

## Introduction and background

As populations age globally, the management of multimorbidity has become increasingly complex and may contribute to inappropriate polypharmacy, commonly defined as the regular use of five or more medications. Among older adults (≥65 years), this escalation in medication burden is a primary driver of adverse drug events (ADEs), drug-drug interactions, and increased healthcare utilization, including preventable hospitalizations [1,2]. To mitigate these risks, deprescribing is understood within the broader framework of medication optimization. It involves the structured, patient-centered review of current therapy to identify medications whose potential harms outweigh benefits, while also recognizing when clinically appropriate therapies may be absent from the regimen [3,4].

Within the multidisciplinary team, clinical pharmacists possess the specialized pharmacotherapeutic expertise required to navigate the complexities of geriatric prescribing [5]. Pharmacist-led models often utilize validated screening tools, most notably the 2023 American Geriatrics Society (AGS) Beers Criteria, to identify potentially inappropriate medications (PIMs) that should be avoided or dose-adjusted in older adults [6]. Furthermore, structured, collaborative pathways such as the Team Approach to Polypharmacy Evaluation and Reduction (TAPER) provide a framework for pharmacists to partner with physicians and patients to reduce medication burden safely [4,7]. In high-risk environments, such as the transition from hospital to community care, interventions like Shed-MEDS - a structured pharmacist-involved transition-of-care protocol - have been developed to ensure prescribing safety is maintained across care settings [8].

Despite the conceptual strength of these models, the current evidence base for pharmacist-led deprescribing remains fragmented. While previous systematic reviews, such as that by Riordan et al., demonstrated that pharmacist-led interventions in primary care could reduce PIMs, the earlier evidence base was limited in quality and predates several recent pharmacist-led deprescribing models and newer syntheses [9]. More recent syntheses have focused on specific settings or multidisciplinary approaches rather than pharmacist-led interventions specifically. For instance, Carollo et al. reported that medication reviews for older inpatients significantly reduced hospital readmissions and PIM counts, yet found no significant impact on mortality, highlighting a gap between process-based success and major clinical outcomes [2]. Similarly, a recent 2025 meta-analysis by Linsky et al. showed that community-based deprescribing interventions may reduce medication burden; however, important questions remain regarding their association with patient safety and health-related quality of life [1].

This lack of clarity is further compounded by methodological heterogeneity across the literature. An overview of systematic reviews by Chua et al. identified inconsistent reporting of clinical outcomes and a reliance on low-quality primary studies, noting that the impact of deprescribing on mortality and hospitalization remains inconclusive [10]. While recent pharmacist-specific reviews have emerged, such as the synthesis by Tesfaye et al., these continue to emphasize medication-related outcomes (e.g., total medication count) rather than the clinical safety and longitudinal well-being of the patient [11].

Consequently, there is a critical need for a high-quality synthesis of recent evidence (2020-2024) that evaluates pharmacist-led deprescribing across the full continuum of care, including hospital, outpatient, and transitions of care. It remains unclear whether the implementation of modern collaborative frameworks, such as TAPER or Shed-MEDS, translates into measurable improvements in patient safety and medication appropriateness. Therefore, this systematic review aims to evaluate the impact of pharmacist-led deprescribing interventions on safety outcomes (ADEs, hospitalizations, and mortality) in older adults, providing an evidence-based assessment of the pharmacist’s role in optimizing geriatric pharmacotherapy.

## Review

Methods

This systematic review was reported according to the Preferred Reporting Items for Systematic Reviews and Meta-Analyses (PRISMA) 2020 statement.

PROSPERO Registration

The review protocol was registered in the PROSPERO database (CRD420251050679). Registration was completed after the literature search had commenced but before final data synthesis. The research question, eligibility criteria, and primary and secondary outcomes were predefined, and no major protocol deviations were introduced during the review process.

Search Strategy

A structured literature search was conducted in PubMed, Embase, and Scopus to identify studies published between January 1, 2020 and December 31, 2024. The search strategy combined controlled vocabulary (where applicable) and free-text terms related to deprescribing, older adults, and pharmacist-led interventions. The search terms included combinations of the following keywords: “deprescribing”, “medication withdrawal”, “medication review”, “older adults”, “elderly”, “geriatric”, “polypharmacy”, “pharmacist-led”, and “clinical pharmacist” (Table 1). To ensure full data extraction and quality appraisal, full texts were retrieved for all potentially eligible studies. Searches were limited to human studies published in English. The final search was completed in December 2024.

**Table 1:** Selected databases, search strategies, and filters applied during the initial article retrieval process.

Database	Search Strategy	Filters applied in the database	Number of records retrieved
PUBMED	("Deprescribing"[Mesh] OR deprescrib*[tiab] OR "medication withdrawal"[tiab] OR "medication discontinuation"[tiab] OR "medication review"[tiab]) AND ("Aged"[Mesh] OR "Frail Elderly"[Mesh] OR elderly[tiab] OR "older adult*"[tiab] OR geriatric*[tiab]) AND ("Pharmacists"[Mesh] OR pharmacist*[tiab] OR "clinical pharmacist*"[tiab])	Publication date: January 1, 2020 to December 31, 2024 Age: 65+ years Document Type: Article Language: English	28
EMBASE	('pharmacist-led':ti,ab OR 'led by pharmacist':ti,ab OR 'clinical pharmacist':ti,ab) AND (deprescribing/exp OR deprescribing) AND ('older adult'/exp OR elderly OR geriatric) AND (polypharmacy/exp OR polypharmacy)	Publication date: January 1, 2020 to December 31, 2024 Age: 65+ years Document Type: Article Language: English	51
SCOPUS	TITLE-ABS-KEY("pharmacist-led" OR "clinical pharmacist" OR pharmacist*) AND TITLE-ABS-KEY(deprescribing OR "medication withdrawal" OR "medication review") AND TITLE-ABS-KEY("older adults" OR elderly OR geriatric) AND TITLE-ABS-KEY(polypharmacy OR "multiple medications") AND TITLE-ABS-KEY("patient safety" OR "adverse drug events")	Publication date: January 1, 2020 to December 31, 2024 Age: 65+ years Document Type: Article Language: English	146

Eligibility Criteria

Eligibility criteria were predefined based on the review question and structured using the PICO framework. The population comprised older adults aged ≥65 years, particularly those who were frail, experiencing polypharmacy, or living with multiple chronic conditions. The intervention included structured deprescribing strategies led by clinical pharmacists, either independently or within multidisciplinary teams, across hospital, outpatient, or transitional care settings. The comparator was usual care without pharmacist-led deprescribing or systematic medication review.

Primary outcomes focused on patient safety, including adverse drug events, hospital readmissions, and all-cause mortality. Secondary outcomes included changes in the prevalence of PIMs, improvements in medication appropriateness, and prescriber acceptance rates of pharmacist recommendations. The review addressed the following question: In older adults aged ≥65 years experiencing polypharmacy or multimorbidity, do pharmacist-led structured deprescribing interventions, compared with usual care, reduce adverse drug events, unplanned readmissions, and all-cause mortality? Additionally, do these interventions improve prescribing appropriateness and reduce potentially inappropriate medication use during follow-up?

Inclusion Criteria: This systematic review included studies published between January 2020 and December 2024 that evaluated pharmacist-led deprescribing interventions (either independently or as part of a multidisciplinary team) in older adults (aged ≥65 years) experiencing polypharmacy or living with multiple chronic conditions. Eligible studies included interventional, quasi-experimental, and retrospective observational designs, provided they reported quantifiable outcomes related to patient safety (such as adverse events, hospital readmissions, or mortality). Only articles published in English were included.

Exclusion Criteria: Opinion pieces, editorials, letters to the editor, systematic reviews, meta-analyses, narrative reviews, and protocols without empirical results were excluded. Likewise, qualitative studies that did not report numerical data on the outcomes of interest were not considered. Prospective studies without a comparison group and reports available only in abstract form were also excluded.

Study Selection

Two investigators independently screened the titles and abstracts of all references retrieved through the database searches to identify potentially relevant studies that met the inclusion criteria. Additionally, backward snowballing of the reference lists of relevant original articles and reviews was conducted to identify any potentially eligible studies missed by the electronic database search. Disagreements between reviewers were resolved through discussion and consensus.

Risk of Bias

Data collection was performed using a pre-designed extraction table developed prior to the evaluation of the selected articles. Two independent reviewers assessed the methodological quality of the eligible studies. Risk of bias was assessed using the Joanna Briggs Institute (JBI) Critical Appraisal Checklist for Randomized Controlled Trials [12] for randomized studies and the JBI Critical Appraisal Checklist for Quasi-Experimental Studies [13] for quasi-experimental studies.

Results

Selection of Studies

**Figure 1:**
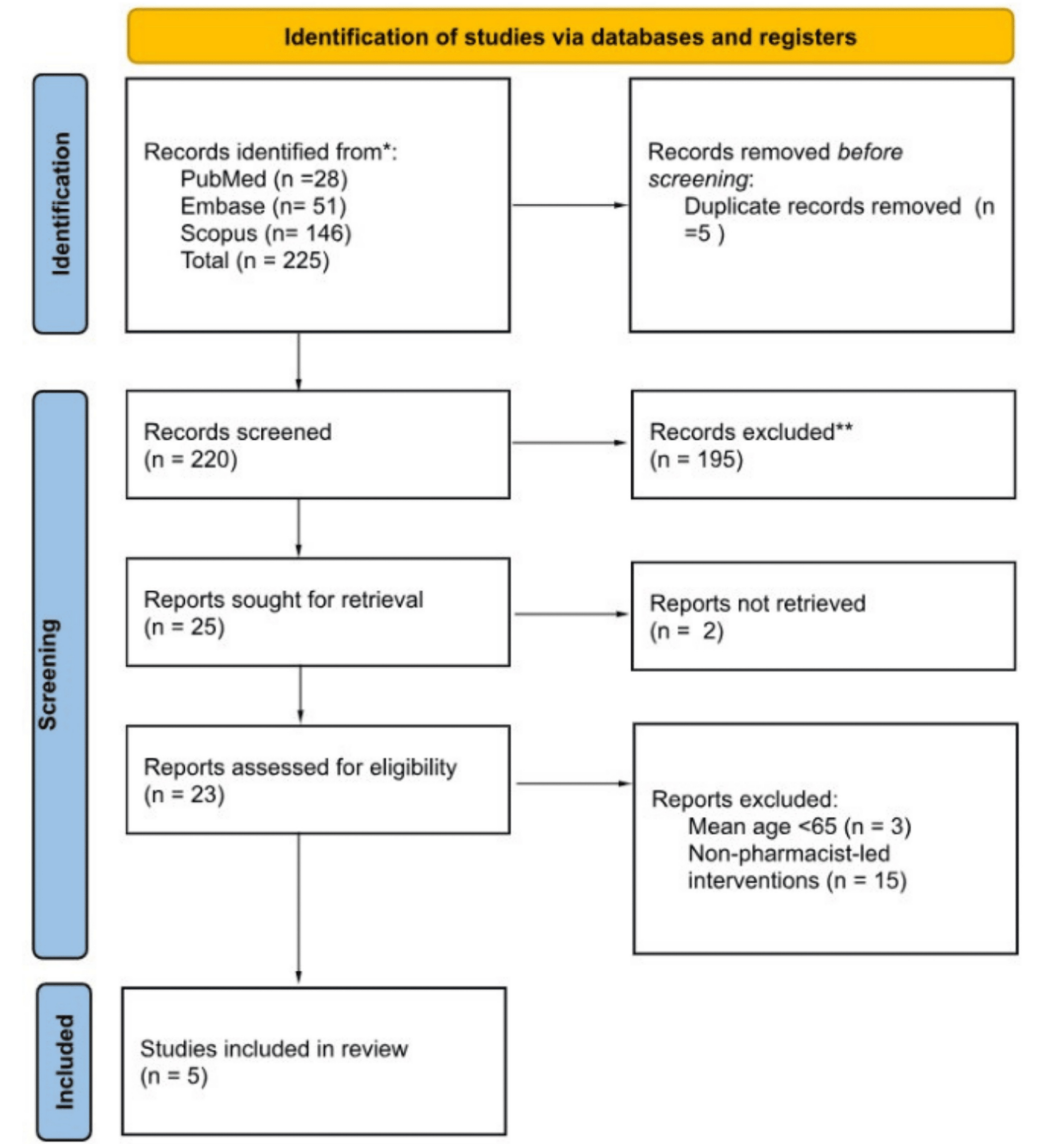
PRISMA 2020 flow diagram of the study selection process. Flow diagram illustrating identification, screening, eligibility assessment, and inclusion of studies evaluating pharmacist-led deprescribing interventions in older adults. The diagram was designed using Canva (Canva Pty Ltd., Sydney, Australia), version 2026. PRISMA: Preferred Reporting Items for Systematic Reviews and Meta-Analyses

A total of 225 records were identified through database searching. After removal of five duplicates, 220 records remained for title and abstract screening. Of these, 195 were excluded for not meeting the inclusion criteria. Twenty-five full-text articles were sought for retrieval, of which two could not be retrieved in full text and were therefore excluded from eligibility assessment. Twenty-three reports were assessed for eligibility, and 18 were excluded for predefined reasons. Ultimately, five studies were included in the qualitative synthesis. The study selection process is summarized in Figure 1.

Characteristics of the Included Studies

The characteristics of the five included studies are summarized in Table 2. The included studies were conducted in Japan, South Korea, the United States, Denmark, and Qatar. The included studies comprised two randomized controlled trials and three quasi-experimental studies, including retrospective pre-post and longitudinal feasibility designs.

**Table 2:** Characteristics of included studies evaluating pharmacist-led deprescribing interventions in older adults. STOPP: Screening Tool of Older Persons’ Prescriptions; START: Screening Tool to Alert to Right Treatment; PIM: potentially inappropriate medications; ADE: adverse drug events; ISAR: Identification of Seniors at Risk tool; PCP: primary care physician; GP: general physician; SFINX: Swedish Finnish Interaction X-referencing; MAI: Medication Appropriateness Index; QTRIM: Qatar Tool for Reducing Inappropriate Medication; TCA: tricyclic antidepressant

Study	Study Design	Country/Setting	Population/Age	N	Follow-up	Intervention	Comparator	Tools Used	Main Findings
Ie et al. [14]	Open-label RCT	Japan / 8 internal medicine inpatient wards	Geriatric inpatients (≥65 years) with polypharmacy (≥5 medications); Mean age: 81.8 years	442 (I=215, C=227)	12 months	Multidisciplinary medication review and optimization proposal; discharge summary sent to PCP and community pharmacist	Usual care, including medication reconciliation	STOPP/START criteria	Significant reduction in PIMs at 12 months (p=0.007); No significant difference was observed in the composite clinical outcome reported.
Lee et al. [15]	Open-label RCT	South Korea / Inpatient (Inha University Hospital)	Geriatric patients (≥65 years) with polypharmacy; Mean age: 73.1 years	32 participants	30 days	Collaborative medication reconciliation, simplification, and patient education	Usual care	Beers; STOPP/START criteria	Significant reduction in ADEs at 30 days (p=0.039); mean acceptance rate of 83%
Jovevski et al. [16]	Quasi-experimental (Retrospective Pre-Post)	USA / Urban Veterans Affairs Emergency Dept.	Patients ≥75 years screening positive on ISAR tool; Mean age: 82 years	298 (Pre=149, Post=149)	60 days	ED pharmacist-led medication reconciliation with deprescribing recommendations sent to PCPs	Historical control (pre-intervention period)	ISAR tool was used to identify high-risk older adults for intervention eligibility rather than to identify PIMs	Significant increase in PIM deprescribing case rate (11.1% to 57.1%, p<0.001)
Houlind et al. [17]	Quasi-experimental (Longitudinal Feasibility)	Denmark / Emergency Department	Geriatric patients (>65 years) with polypharmacy (≥5 medications); Median age: 80 years	60 enrolled (39 completed)	30 days (180 days for readmission)	Collaborative medication review (pharmacist + geriatrician) with recommendations to PCP/GP	Quasi-experimental retrospective pre-post study comparing outcomes in the pre-intervention and post-intervention periods	STOPP; SFINX; Renbase	Significant reduction in median MAI score (14 to 8, p<0.001) at 30 days
Alyazeedi et al. [18]	Quasi-experimental (Quality Improvement)	Qatar / Ambulatory (Geriatric and Dermatology clinics)	Ambulatory older adults; Mean age: 71.4 years	155 prescriptions reviewed	14 months (total study period)	Implementation of QTRIM (pharmacist-led) to identify and reduce PIMs	Pre-implementation period	QTRIM (Beers-based tool)	Significant reduction in specific PIM classes (TCAs, antihistamines)

The clinical settings were heterogeneous, encompassing acute inpatient wards (n = 2), emergency departments (n = 2), and ambulatory clinics (n = 1). The study populations consistently targeted older adults, with mean or median ages ranging from 71.4 to 82 years, characterized by polypharmacy, typically defined as the use of five or more regular medications.

To identify PIMs, the interventions utilized validated screening tools. The Screening Tool of Older Persons’ Prescriptions (STOPP) criteria was the most frequently applied instrument (n = 5), often used in conjunction with the Beers Criteria (n = 4) or locally developed tools such as the Qatar Tool for Reducing Inappropriate Medication (QTRIM), used to identify potentially inappropriate medications in ambulatory older adults. Detailed information regarding study design, intervention components, comparators, follow-up duration, screening tools, and principal findings is provided in Table 2.

Primary and Secondary Outcomes

The effects of pharmacist-led interventions on medication appropriateness and safety outcomes are summarized in Table 3. Regarding the primary safety outcome of adverse drug events, only one study reported a statistically significant reduction. Lee et al. [15] observed zero events in the intervention group compared with five in the control group at 30 days post-discharge (p = 0.039); however, this finding should be interpreted cautiously, given the small sample size of the trial (n = 32), which limits statistical precision and may result in unstable event estimates. In the largest randomized trial, Ie et al. [14] reported that the incidence of adverse events was similar between groups (57.2% in the intervention group vs. 59.5% in usual care), and no significant difference was observed in the composite clinical outcome reported in the original study (mortality, rehospitalization, and unscheduled visits). Similarly, the quasi-experimental study by Jovevski et al. [16] found no significant changes in emergency department visits, hospitalizations, or mortality at 60 days following the intervention.

**Table 3:** Effects of pharmacist-led deprescribing interventions in older adults: reduction of potentially inappropriate medications and adverse drug events by follow-up period. STOPP: Screening Tool of Older Persons’ Prescriptions; START: Screening Tool to Alert to Right Treatment; PIM: potentially inappropriate medications; MRCI-K: Korean version of the Medication Regimen Complexity Index; AOU: Assessment of Underutilization Index

Study	Type of Intervention	Follow-up Period	Reduction of PIMs	Reduction of Adverse Events
Lee et al. [15]	Pharmacist-led comprehensive medication reconciliation	30 days post-discharge	Medication reviews conducted using Beers and STOPP/START criteria. Regimen complexity (MRCI-K) decreased by a mean of 6.2 points in the intervention group vs. 2.4 points in the control group (p=0.159)	0 events in the intervention group vs. 5 events in the control group (p=0.039) at 30 days.
Ie K. et al. [14]	Multidisciplinary team-based medication optimization protocol	12 months	Significant reduction in patients with ≥1 PIM at discharge (26.2% vs 33.0%, p=0.03) and at 12 months (26.7% vs 37.4%, p=0.007)	Adverse events were similar: 57.2% (intervention) vs. 59.5% (usual care). No significant difference in composite clinical outcome
Alyazeedi A. et al. [18]	QTRIM (Qatar Tool for Reducing Inappropriate Medication)	14 months	Significant reductions in specific PIMs: 66.6% in tricyclic antidepressants, 51.7% in 1st gen antihistamines, and 33.3% in muscle relaxants.	Not reported. The study focused on PIM reduction as the primary outcome measure
Houlind M.B. et al. [17]	Collaborative medication review (pharmacist + geriatrician)	30 days post-discharge	AOU score ≥1 was reduced from 36% to 10% (p<0.001).	Not reported. The study evaluated feasibility and sustainment of medication changes
Jovevski J.J. et al. [16]	ED pharmacist-led medication reconciliation protocol	60 days	Deprescribing case rate increased from 11.1% (pre-intervention) to 57.1% (post-intervention) (p<0.001). 49% of PIMs remained unchanged vs 91% pre-intervention (p<0.05)	No significant improvement in 7- or 30-day subsequent ED visits, hospitalizations, or 60-day mortality.

Reductions in the prevalence or burden of PIMs were consistently reported across the five studies with available data. In the trial by Ie et al. [14], the proportion of patients with one or more PIMs was significantly lower in the intervention group at discharge (26.2% vs. 33.0%, p = 0.03) and at the 12-month follow-up (26.7% vs. 37.4%, p = 0.007). Using the Medication Appropriateness Index (MAI), Houlind et al. [17] observed a significant decrease in median scores from 14 (IQR 8-20) to 8 (IQR 2-13) at 30 days (p < 0.001). Targeted reductions were also observed in ambulatory settings, where Alyazeedi et al. [18] reported substantial decreases in the prescription of high-risk classes, including tricyclic antidepressants (66.6%) and first-generation antihistamines (51.7%). Furthermore, Jovevski et al. [16] demonstrated that a pharmacist-led protocol increased the case rate of PIM deprescribing from 11.1% in the pre-intervention period to 57.1% post-intervention (p < 0.001).

Risk of Bias Assessment

**Figure 2:**
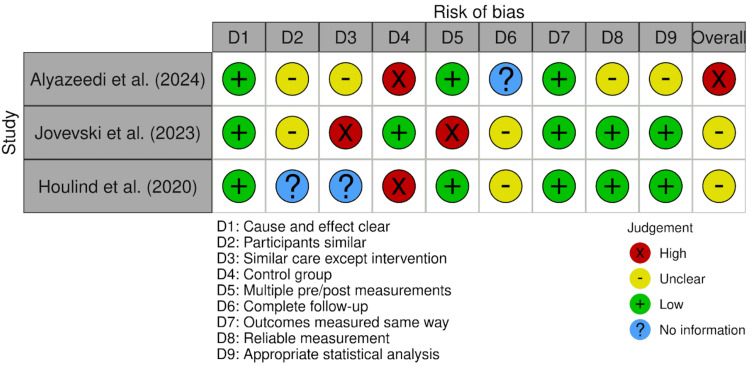
Methodological quality assessment of included quasi-experimental studies according to the JBI Critical Appraisal Tool. The diagram was designed using Canva (Canva Pty Ltd., Sydney, Australia; 2026 version) based on the Joanna Briggs Institute (JBI) Critical Appraisal Framework [16-18]. JBI Critical Appraisal Framework by Joanna Briggs Institute (Adelaide, Australia) [13].

Assessment Using the JBI Critical Appraisal Tool for Quasi-Experimental Studies: The methodological quality of the included quasi-experimental studies was assessed using the Joanna Briggs Institute (JBI) critical appraisal tool [13]. The detailed risk of bias assessment across the nine domains is presented in Figure 2.

Overall, all quasi-experimental studies clearly defined the intervention and outcome measures. However, important methodological limitations were identified. These included the absence of control groups in some studies, incomplete reporting of participant comparability, and limited detail regarding follow-up procedures. In addition, concerns were noted in specific domains such as multiple pre-post measurements and potential confounding factors. These methodological limitations may affect the internal validity of the findings and should be considered when interpreting the reported intervention effects.

**Figure 3:**
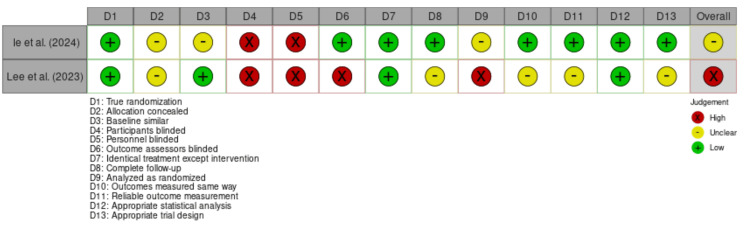
Methodological quality assessment of the included randomized controlled trials according to the JBI Critical Appraisal Tool. The diagram was designed using Canva (Canva Pty Ltd., Sydney, Australia; 2026 version) based on the Joanna Briggs Institute (JBI) Critical Appraisal Framework for randomized controlled trials [14,15]. JBI Critical Appraisal Framework by Joanna Briggs Institute (Adelaide, Australia) [12].

Assessment Using the JBI Critical Appraisal Tool for Randomized Controlled Trials: The methodological quality of the included randomized controlled trials was evaluated using the JBI Critical Appraisal Tool for RCTs [12]. The detailed assessment across all appraisal domains is presented in Figure 3. Both randomized controlled trials reported randomization procedures. However, the specific method of sequence generation was not consistently described in sufficient detail across studies. Allocation concealment was incompletely reported. Although blinding is often impractical in pharmacist-led interventions, the absence of participant and outcome assessor blinding may still increase the risk of performance and detection bias, particularly for non-objective outcomes. In one study, post-randomization exclusions and lack of blinded outcome assessment further threatened internal validity. These methodological limitations should be considered when interpreting the reported intervention effects, particularly regarding safety outcomes and composite clinical endpoints.

Discussion

Clinical Impact of Pharmacist-Led Interventions on Deprescribing and Patient Safety

The findings of this systematic review indicate that pharmacist-led interventions were generally associated with reductions in the burden of potentially inappropriate medications across multiple care settings. Evidence from the randomized controlled trial by Ie et al. [14] and the quasi-experimental studies by Jovevski et al. [16] and Alyazeedi et al. [18] demonstrates consistent success in identifying and discontinuing high-risk medications. Notably, the Japanese randomized controlled trial by Ie et al. [14] showed a sustained reduction in PIM prevalence at 12 months, suggesting that such interventions may have sustained effects on prescribing patterns; however, evidence on long-term durability remains limited because only one included study reported follow-up at 12 months. 

However, the impact on major clinical outcomes remains less definitive. Lee et al. [15] reported a statistically significant reduction in ADEs within 30 days post-discharge (0 vs. 5 events, p = 0.039). However, this finding should be interpreted cautiously because the trial included only 32 participants, limiting statistical power and the precision of event estimates. The larger trial by Ie et al. [14] found no significant difference in a composite outcome of mortality, rehospitalization, and unscheduled visits. Similarly, Jovevski et al. [16] observed no changes in ED revisits or mortality despite a substantial increase in the deprescribing case rate from 11.1% to 57.1%. This suggests that while pharmacist-led protocols are effective at optimizing medication lists and did not demonstrably increase adverse clinical outcomes across the included studies, it should be noted that none of the included trials were powered or designed as formal safety studies. The absence of increased harm should therefore be interpreted as reassuring rather than as definitive evidence of safety.

Although not included in the present review, Moga et al. [19] demonstrated that a pharmacist-physician collaborative medication therapy management intervention significantly improved prescribing quality and reduced anticholinergic burden in community-dwelling older adults. The intervention led to measurable improvements in the MAI and reductions in Anticholinergic Drug Scale (ADS) scores, which may indicate decreased exposure to high-risk medications. Importantly, these improvements were achieved without deterioration in patient-reported health outcomes over the follow-up period. This evidence further supports the clinical impact of pharmacist-led deprescribing strategies, highlighting their ability to enhance medication safety while maintaining overall patient stability. 

Feasibility, Acceptability, and the Role of the Pharmacist

A key finding across the included studies is the high level of feasibility and professional acceptance of pharmacist-led deprescribing. In the South Korean randomized controlled trial, Lee et al. [15] reported that the acceptance rate of pharmacist recommendations reached 83%, while the Danish feasibility study by Houlind et al. [17] reported that 60% of recommendations were implemented. These findings suggest that pharmacist recommendations were frequently accepted within collaborative care models involving physicians or geriatricians.

The diverse settings of these studies, ranging from emergency departments to specialized ambulatory clinics, underscore the adaptability of the pharmacist’s role. Jovevski et al. [16] and Houlind et al. [17] evaluated interventions implemented in emergency department settings, whereas Alyazeedi et al. [18] reported outcomes from specialized ambulatory clinics. In the emergency department setting, pharmacists can act as critical gatekeepers who initiate the deprescribing process during acute transitions of care, a period often associated with high medication instability [7].

Elshazly et al. [20] highlighted key factors such as lack of experience and awareness regarding the deprescribing process, low perceived self-efficacy among healthcare professionals, and concerns about potential clinical consequences of discontinuing medications. Additional obstacles include poor communication and collaboration among pharmacists, patients, and other healthcare team members, as well as a fragmented healthcare system that limits appropriate clinical information sharing. Furthermore, institutional disincentives, resource constraints (including time), insufficient financial support, and persistent hierarchical dynamics that position physicians as dominant decision-makers can undermine the active and autonomous participation of pharmacists and nurses in shared clinical decisions. 

Identification of Implementation Barriers

Despite high acceptance rates, several barriers to the effective implementation of deprescribing were identified or implied within the available data. Time and resource constraints remain significant. The feasibility study conducted by Houlind et al. [17] reported an attrition rate of 35.0% (21 of 60 enrolled participants did not complete follow-up), reflecting the challenges of maintaining follow-up in acute care settings. Furthermore, Jovevski et al. [16] observed that 49% of identified potentially inappropriate medications remained unchanged after the intervention, indicating that the denominator refers to medications rather than patients and suggesting that clinical inertia or prescriber reluctance may persist even when pharmacists provide evidence-based recommendations 

**Figure 4:**
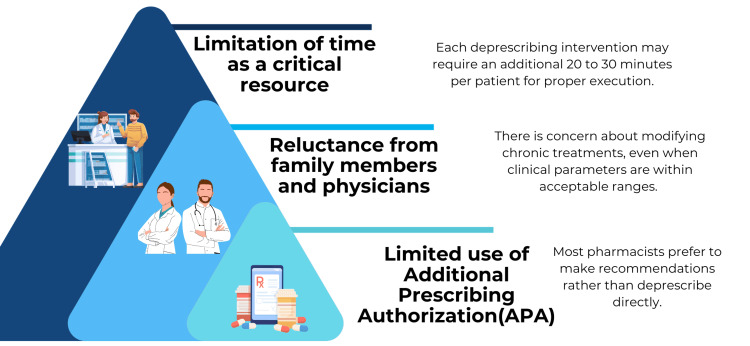
Main barriers identified for the effective implementation of pharmacist-led deprescribing interventions. Graphical representation of key structural and professional barriers to pharmacist-led deprescribing, including time limitations, resistance from family members and physicians, and limited use of Additional Prescribing Authorization (APA). The conceptual framework is based on findings reported by Vucenovic et al. [21]. The figure was manually designed by the authors using Canva (Canva Pty Ltd., Sydney, Australia) and does not contain generative AI–generated content.

Although not eligible for inclusion due to the predefined search cut-off (December 2024), a recent study by Vucenovic et al. [21] identified three main barriers to pharmacist-led deprescribing, as depicted in Figure 4, which align with the challenges previously discussed. The first barrier is the underutilization of Additional Prescribing Authorization (APA), as many pharmacists prefer to make recommendations rather than deprescribe directly, often due to professional insecurities or concerns about negative responses from physicians. The second barrier is resistance from family members and physicians, particularly regarding the withdrawal of medications such as antihypertensives, even when patients’ blood pressure levels are within acceptable ranges. Finally, the third barrier relates to time limitations, as each deprescribing intervention is estimated to require an additional 20 to 30 minutes per patient to be properly executed.

Future Research Directions

There is a clear need for future research to bridge the gap between intermediate outcomes (PIM reduction) and patient-centered clinical endpoints. Although not included in the present review due to the predefined eligibility criteria, Mangin et al. [7] noted in their protocol that future trials should prioritize health-related quality of life and functional status as primary outcome measures. Moreover, the long-term sustainability of deprescribing, specifically whether PIMs are restarted after discharge, requires further investigation, as only one study in this review provided follow-up data beyond six months (Ie et al. [14]). Future studies should also explore the economic impact and cost-effectiveness of integrating geriatric pharmacists into standard care teams.

Limitations of This Review

This review is limited by the small number of included studies (n = 5) and significant heterogeneity in study design, clinical settings, intervention components, follow-up duration, and reported outcomes. The inclusion of three quasi-experimental studies, which lack control groups or randomization, increases the risk of bias and limits the ability to draw causal conclusions. Finally, the open-label nature of the randomized controlled trials conducted by Ie et al. [14] and Lee et al. [15] may introduce performance bias, particularly for non-objective outcomes.

## Conclusions

Pharmacist-led deprescribing interventions appear feasible and were generally associated with reductions in potentially inappropriate medication use and improved medication appropriateness. Evidence regarding major clinical outcomes remains limited and heterogeneous, and current data do not establish a definitive benefit for hospitalization, mortality, or other patient-centered outcomes. Larger, adequately powered randomized studies with longer follow-up are needed.
